# Identification and validation of a novel prognostic model of inflammation-related gene signature of lung adenocarcinoma

**DOI:** 10.1038/s41598-022-19105-8

**Published:** 2022-08-30

**Authors:** Dayuan Luo, Wei Feng, Yunqian Ma, Zhibin Jiang

**Affiliations:** Department of Cardiothoracic Surgery, Xiangya Third Hospital, Central South University, Changsha, 410013 Hunan China

**Keywords:** Cancer, Cell biology, Computational biology and bioinformatics, Genetics, Immunology, Biomarkers, Diseases, Medical research, Molecular medicine, Oncology, Risk factors

## Abstract

Previous literatures have suggested the importance of inflammatory response during lung adenocarcinoma (LUAD) development. This study aimed at exploring the inflammation-related genes and developing a prognostic signature for predicting the prognosis of LUAD. Survival‑associated inflammation-related genes were identified by univariate Cox regression analysis in the dataset of The Cancer Genome Atlas (TCGA). The least absolute shrinkage and selection operator (LASSO) penalized Cox regression model was used to derive a risk signature which is significantly negatively correlated with OS and divide samples into high-, medium- and low-risk group. Univariate and multivariate Cox analyses suggested that the level of risk group was an independent prognostic factor of the overall survival (OS). Time-dependent receiver operating characteristic (ROC) curve indicated the AUC of 1-, 3- and 5-years of the risk signature was 0.715, 0.719, 0.699 respectively. A prognostic nomogram was constructed by integrating risk group and clinical features. The independent dataset GSE30219 of Gene Expression Omnibus (GEO) was used for verification. We further explored the differences among risk groups in Gene set enrichment analysis (GSEA), tumor mutation and tumor microenvironment. Furthermore, Single Sample Gene Set Enrichment Analysis (ssGSEA) and the results of Cell-type Identification By Estimating Relative Subsets Of RNA Transcripts (CIBERSORT) suggested the status of immune cell infiltration was highly associated with risk groups. We demonstrated the prediction effect of CTLA-4 and PD-1/PD-L1 inhibitors in the low-risk group was better than that in the high-risk group using two methods of immune score include immunophenoscore from The Cancer Immunome Atlas (TCIA) and TIDE score from Tumor Immune Dysfunction and Exclusion (TIDE). In addition, partial targeted drugs and chemotherapy drugs for lung cancer had higher drug sensitivity in the high-risk group. Our findings provide a foundation for future research targeting inflammation-related genes to predictive prognosis and some reference significance for the selection of immunotherapy and drug regimen for lung adenocarcinoma.

## Introduction

Lung carcinoma has the highest mortality among all types of malignant tumors and is the second most common cancer worldwide, which is divided into small cell lung cancer (SCLC) and non-small cell lung cancer (NSCLC) according to cell origin^1^. Adenocarcinoma is the most common type of all newly diagnosed NSCLC cases^2^. The inflammatory response of lung adenocarcinoma runs through all stages of lung tumor development and has an inseparable relationship with its prognosis^3^. Chronic inflammation may lead to tumorigenesis by inducing gene mutations, cell proliferation, anti-apoptosis, enhanced invasion, promotion of angiogenesis and secretion of immunosuppressive factors. Inflammatory cytokines and chemokines secreted during lung inflammation, such as tumor necrosis factor (TNF), transforming growth factor-β (TGF-β), Interleukin-1 (IL-1) and cyclooxygenase-2 (COX-2) have been shown to be related to the occurrence and development of lung cancer^4–6^. In addition, immune and microenvironmental disorders associated with inflammatory responses are also thought to be contributing factors to cancer. Tumor microenvironment (TME), which is composed of tumor cells, immune cells, stromal cells, inflammatory mediators and extracellular matrix, participates in the proliferation, drug resistance and metastatic growth of tumor cells together with immune disorders^7,8^.

In view of the close relationship among inflammatory response, immune and tumor microenvironment, inflammation is an important part of lung tumor research. At present, there are many basic studies on inflammatory environment of lung cancer, but few clinical translational studies, especially few studies on inflammation-related genes as prognostic indicators. In the present study, we constructed an accurate 8-gene predictive model and identified a prognostic risk signature for LUAD patients using inflammation-related genes. Our findings indicate the potential connection between the risk signature, prognosis, the immune microenvironment, tumor mutation, immunotherapy and drug sensitivity of LUAD patients.

## Results

### Prognostic model of inflammation-related genes

**Figure 1:**
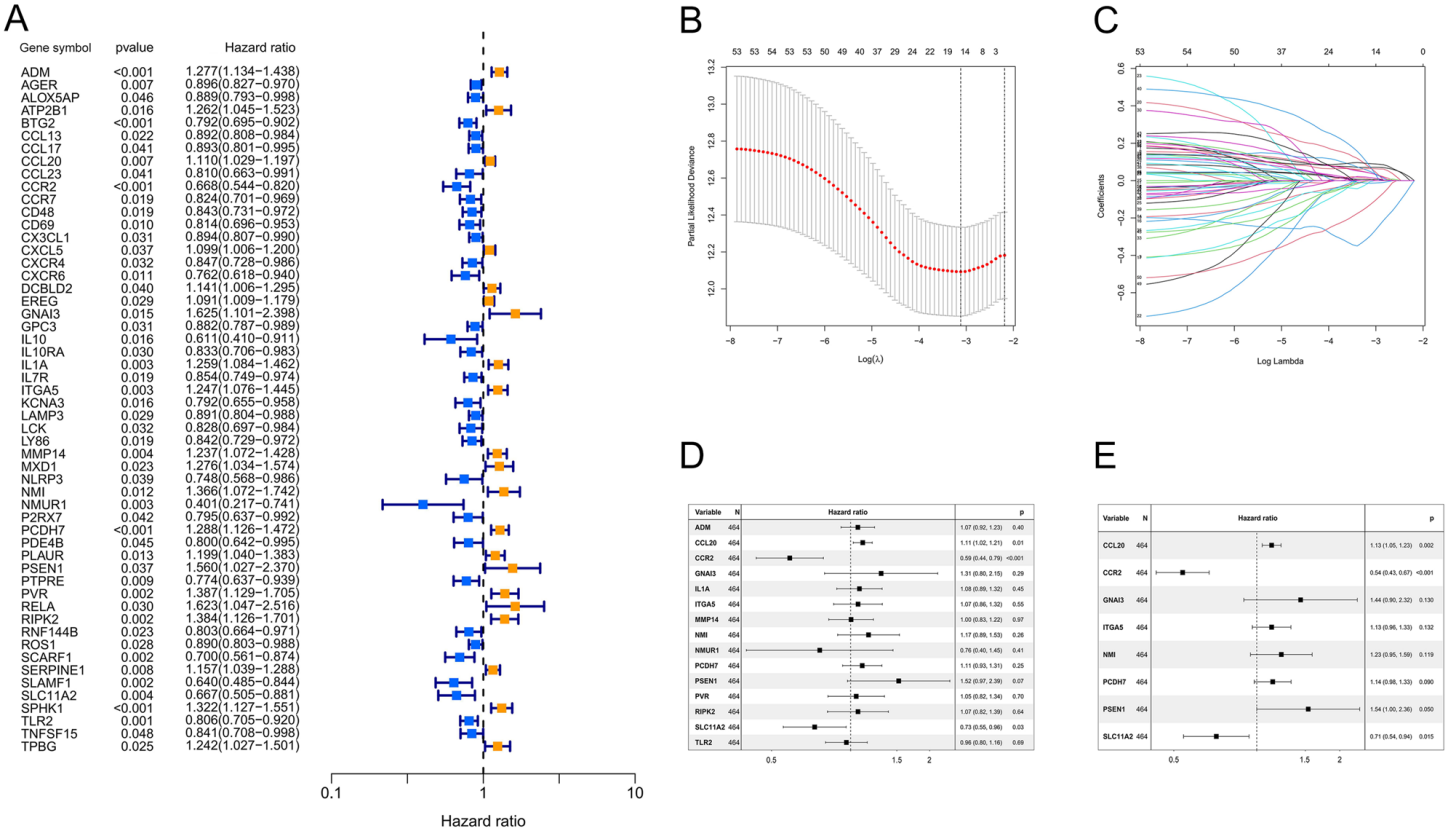
Univariate Cox, LASSO and multivariate Cox regression analysis for overall survival related inflammation-related genes using R software. (**A**) Forest plots showing the prognostic value detection of inflammation-related genes, in which the HRs, corresponding 95% confidence intervals, and *p*-values are displayed. (**B**) LASSO regression analysis was used to calculate the coefficient of inflammation-related genes. (**C**) Fifteen genes were selected as active covariates to determine the prognostic value after ten-fold cross-validation for the LASSO model. (**D**) Forest plot showing the fifteen genes of the LASSO model. (**E**) Forest plot showing the Eight genes which were selected by stepwise forward and backward regression methods for the Cox proportional hazard model.

The flow chart of this study was presented (Supple Fig. 1). 551 samples (54 none-tumor samples and 497 tumor samples) of LUAD were downloaded from TCGA. Excluding none-tumor samples and samples with incomplete information of survival time and survival status, 464 eligible samples were obtained. 254 inflammation-related genes were obtained from Molecular Signatures Database (MSigDB). R-package "survival" was used for univariate Cox analysis to obtain the prognostic genes. As shown in the forest map (Fig. 1A), 54 inflammation-related genes associated with OS were obtained. LASSO regression is used to perform variable screening and re-sampling 1000 times for these 54 genes, and selected the genes with more than 900 repetitions. The figure shows that the optimal number of variables is equal to 15 (Fig. 1B–D). We further conducted multivariate Cox regression analysis on these 15 genes by stepwise method. According to Akaike Information Criterion (AIC), eight prognostic inflammation-related genes were included in the Cox proportional hazard model when the AIC value is the minimum (AIC = 1802.5) (Fig. 1E).

### Validation of risk groups and genetic prognostic models

**Figure 2:**
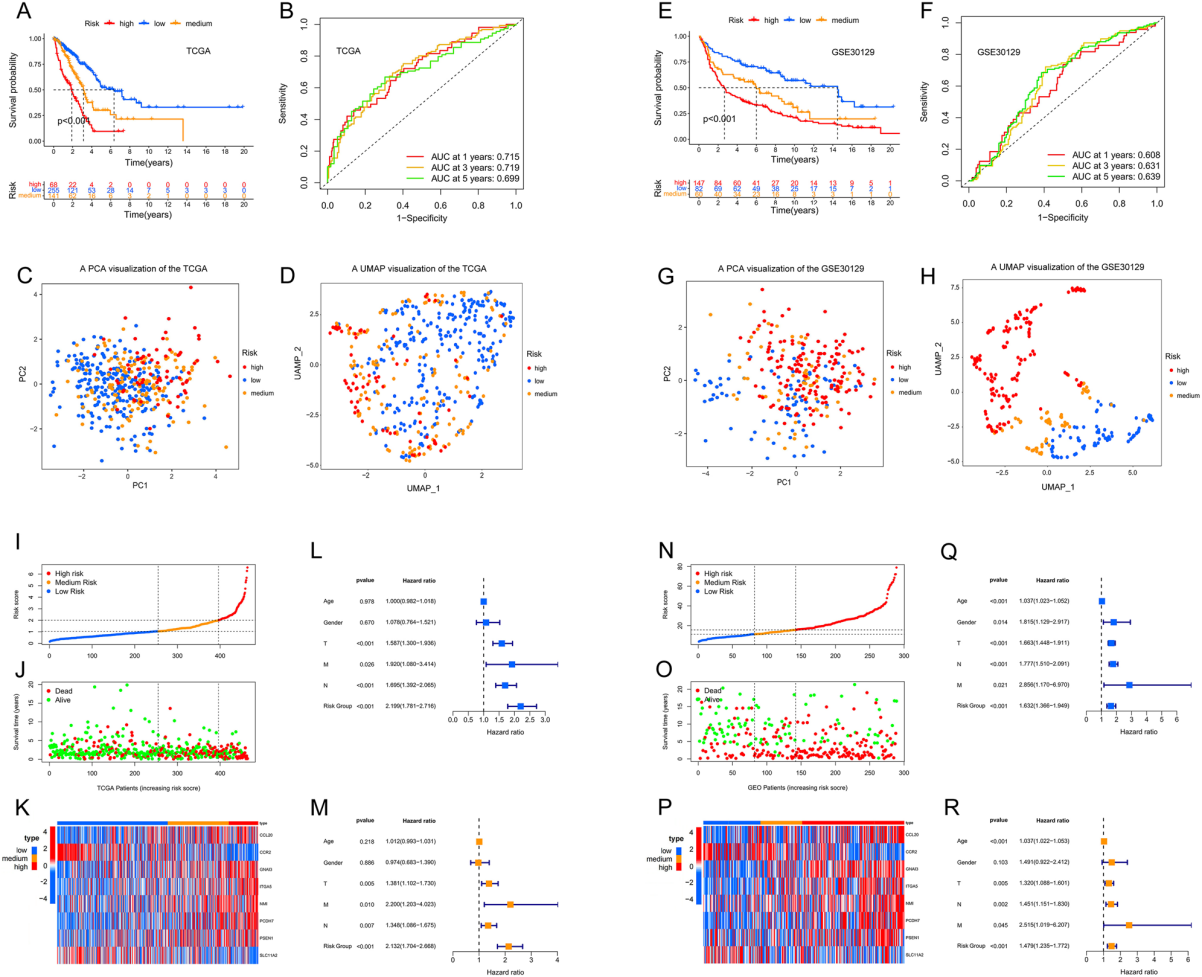
Identification and validation of the prognostic eight-gene risk signature in the TCGA cohort and GSE30219 using R software. (**A**,**E**) Kaplan–Meier curves of overall survival probability of risk groups in TCGA (**A**) and GSE30219 (**E**). (**B**,**F**) Kaplan–Meier overall survival rate curves for the three groups in TCGA (**B**) and GSE30219 (**F**). (**C**,**G**) PCA in the TCGA cohort (**C**) and GSE30219 (**G**). (**D**,**H**) Umap analysis in the TCGA cohort (**D**) and GSE30219 (**H**). (**I**,**N**) Risk score distribution in the TCGA cohort (**I**) and GSE30219 (**N**). (**J**,**O**) Survival time and survival status distribution in the TCGA cohort (**J**) and GSE30219 (**O**). (**K**,**P**) Heatmap showing the expression level for eight inflammation-related genes among the risk groups in TCGA (**K**) and GSE30219 (**P**). (**L**,**Q**) Univariate Cox regression analysis of the association between clinical characteristics, the risk score, and patient overall survival in the TCGA cohort (**L**) and GSE30219 (**Q**). (**M**,**R**) Multivariate Cox regression analysis of the association between clinical characteristics, the risk score, and patient overall survival in the TCGA cohort (**M**) and GSE30219 (**R**).

**Figure 3:**
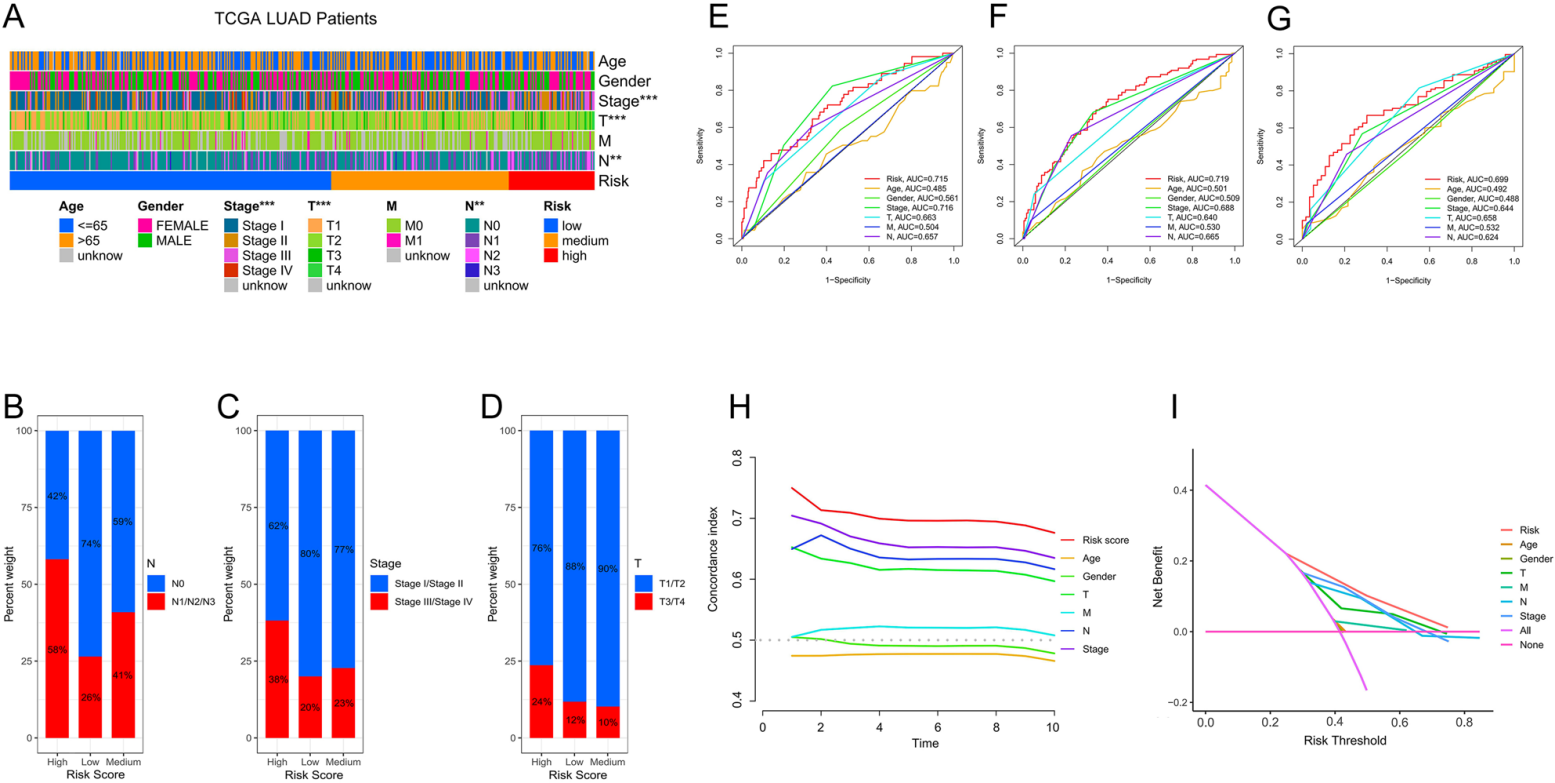
Correlations analysis between the risk signature and clinical features using R software. (**A**) The heatmap and clinical features of three risk groups. (**B**–**D**) Bar plot of correlation between risk score and N (**B**), Stage (**C**), T (**D**) of LUAD. (**E**–**G**) ROC curves for 1-year (**E**), 3-year (**F**), 5-year (**G**) survival prediction and clinical characteristics. (**H**) Time-related concordance index of risk score and clinical characteristics. (**I**) The decision curve analysis of risk score and clinical characteristics. *p-value < 0.05, **p-value < 0.01, ***p-value < 0.001.

Regression coefficients of eight genes included in the prognostic model are shown (Supple Table 1). The risk score of each sample is calculated as follows formula: Risk Score = 0.1241*CCL20 expression − 0.6192*CCR2 expression + 0.3660*GNAI3 expression + 0.1240*ITGA5 expression + 0.2050*NMI expression + 0.1330*PCDH7 expression + 0.430*PSEN1 expression − 0.3382*SLC11A2 expression. The two best cut-off values of risk score are equal to 1.05, 2.01 respectively, the tumor samples were divided into high-, medium- and low-risk group (Supple Fig. 2). Excluding samples with incomplete information of survival time and survival status, a total of 289 tumor samples of GSE30219 from GEO were taken as the validation group, risk score and risk group were defined as the same way, the two best cut-off values of risk score are equal to 11.28 and 15.7 (Supple Fig. 3). Survival analysis based on R-package “survival” showed that the overall survival differences were statistically significant among the three risk groups both in the two data sets(*p*-value < 0.001) (Fig. 2A,E). R-package “timeROC” was used to construct time-dependent ROC curves. AUC at 1-, 3- and 5-years was equal to 0.715, 0.719, 0.699 in the cohort of TCGA (Fig. 2B), and 0.608, 0.631, 0.639 in GSE30219, which shows that the model has good predictive ability (Fig. 2F). Principal component analysis (PCA) and uniform manifold approximation and projection analysis (UMAP) indicate that all samples can be well clustered as risk groups (Fig. 2C,D,G,H). Univariate and multivariate analysis suggested that risk group can serve as an independent prognostic signature of LUAD both in TCGA cohort and GSE30219 (Fig. 2L,Q,M,R). The survival state curve shows most of the survival cases are concentrated in the low-risk group, while the death cases tend to be concentrated in the high-risk group, indicates there is a significant correlation between the risk score and the survival status of the patients (Fig. 2I,J,N,O).

### Clinical correlation of the prognostic factor

The clinical baseline characteristics of TCGA cohort (Supple Table 2) and GSE30219 (Supple Table 3) are shown. The risk score was significantly correlated with the clinical features such as tumor stage (*p*-value < 0.001), T (*p*-value < 0.001) and N (*p*-value < 0.01) (Fig. 3A), the proportion of samples of later tumor stage, T and N tend to be higher as the level of risk group raise up (Fig. 3B–D). The decision curve analysis shows that the net benefit rate of risk score is the highest at each threshold probability compared with other clinical characteristics, suggesting that it have practical application value (Fig. 3I). The ROC curve constructed by clinical characteristics showed the AUC of risk score and tumor stage in predicting 1-year survival was 0.715 and 0.716, which were close to each other. The AUC of 3-year and 5-year survival predicted by risk score was 0.719 and 0.699, higher than 0.688 and 0.644 of 3-year and 5-year survival predicted by tumor stage (Fig. 3E–G). The C-index of risk score was higher than that of other clinical characteristics in univariate regression model with bootstrap re-sampling (1000 times) method (Fig. 3H).

### Construct and verify the prognostic nomogram

**Figure 4:**
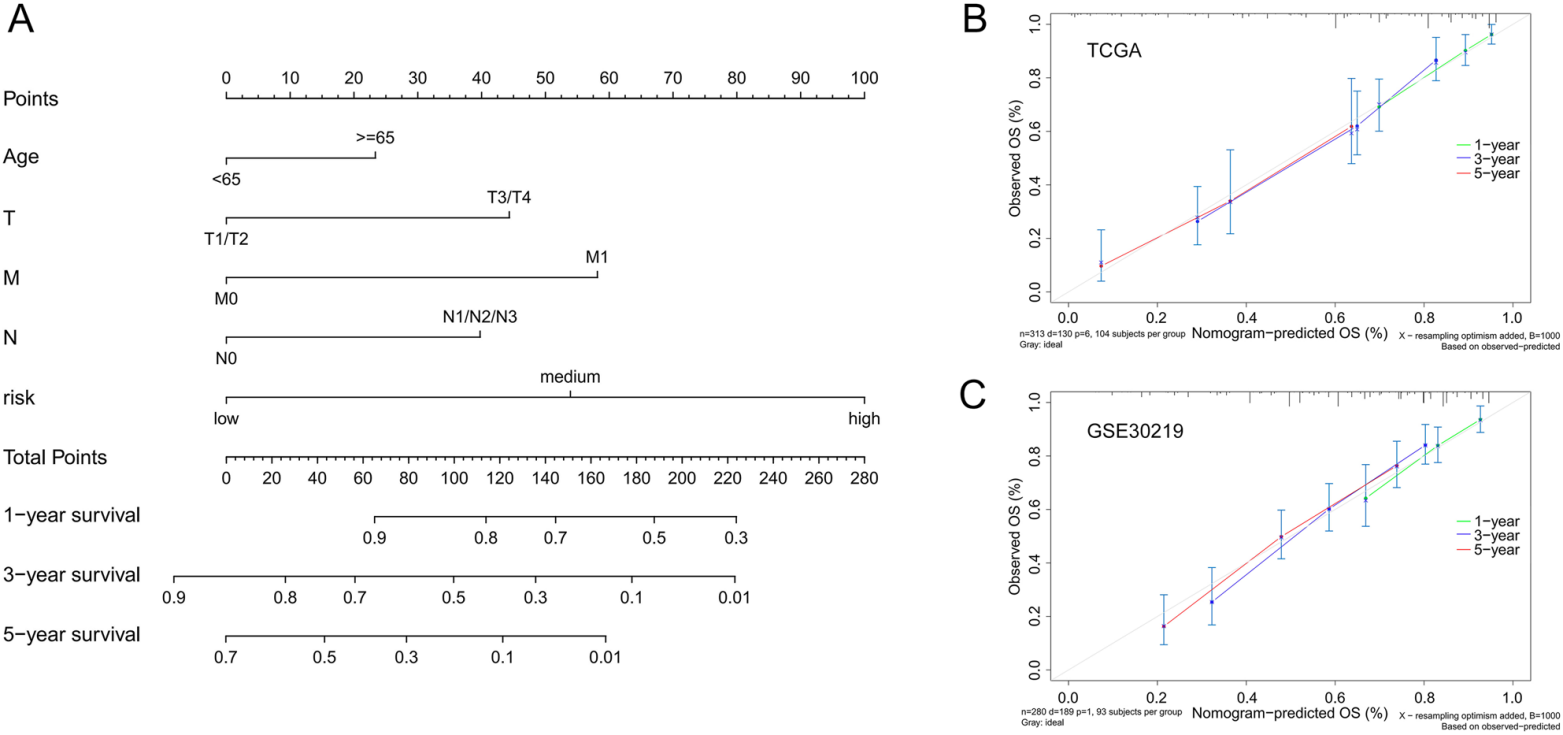
Construction and verification of the Nomogram using R software. (**A**) Nomogram of TCGA cohorts based on the risk groups and clinical features. (**B**) 1-, 3-, 5-year calibration curve for verification of nomogram in the TCGA cohort. (**C**) 1-, 3-, 5-year calibration curve for verification of nomogram in GSE30219.

We integrated signatures including risk groups, T, M, N and age into the Cox proportional hazard model to construct a nomogram (Fig. 4A). According to the clinical factors included in the nomogram, the sample should contain the following 6 kinds of information: overall survival, survival status, T, M, N, and age. Samples with any of the above data missing were excluded. The number of eligible samples in TCGA and GEO cohort was 313 and 280, respectively. The C-index of the nomogram in the TCGA cohort and GSE30219 are equal to 0.745 and 0.692. According to the calibration curves (Fig. 4B,C), the prediction probability of this nomogram is very close to the observation probability after 1000 simulations of the 1-, 3- and 5-years calibration curves drawn by bootstrap re-sampling method in the TCGA cohort and GSE30219.

### Gene set enrichment analysis

**Figure 5:**
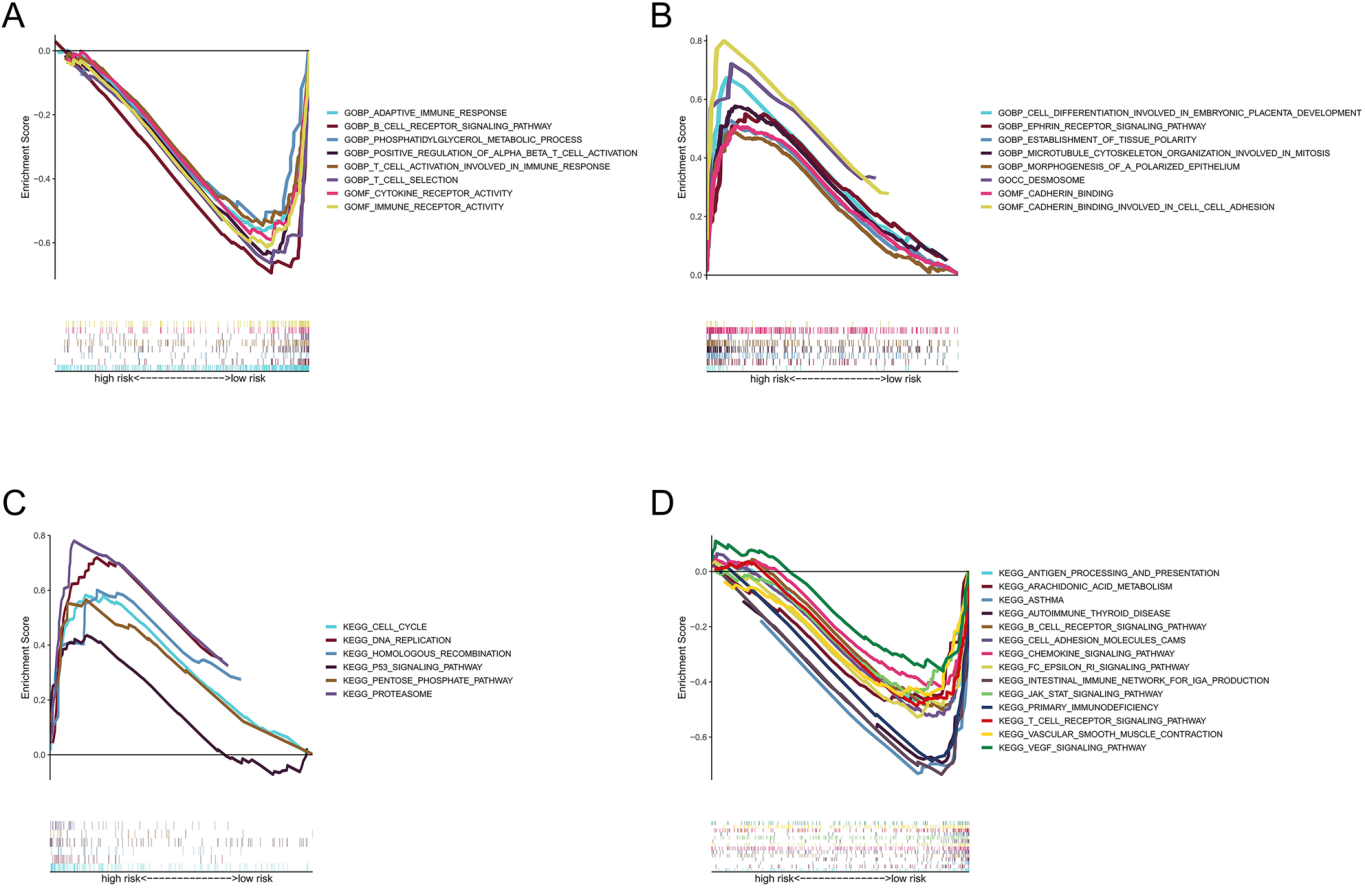
Gene Set Enrichment Analysis of the mRNAs associated with the high or low risk group in TCGA using GSEA software. (**A**) Top enriched GO functions in the low-risk group. (**B**) Top enriched GO functions in the high-risk group. (**C**) Top enriched KEGG pathways in the high-risk group. (**D**) Top enriched KEGG pathways in the low-risk group. The names of enriched KEGG pathways or GO functions are listed on the right side (*p*-value < 0.05).

Gene set enrichment analysis (GSEA) was carried out on the high- and low-risk group in TCGA by using GSEA software (version 4.1.0). In terms of KEGG pathways, the high-risk group mainly focused on cell cycle regulation, DNA replication, homologous chromosome, p53 signaling pathway, pentose phosphate pathway, etc. In the low-risk group, the main pathways were enriched in T and B cell receptor signal pathway, cell adhesion signal pathway, chemokine signal pathway, etc. In terms of GO functions, the high-risk group mainly focused on mitosis, epithelial polarization, protein regulation, desmosome, cadherin, etc. The low-risk group focused on adaptive immune response, B cell receptor signal transduction, lipid metabolism, immune-related T cell activity, T cell selectivity, cytokine receptor activity, immune checkpoint activity (Fig. 5), etc.

### Tumor microenvironment and tumor mutation burden

**Figure 6:**
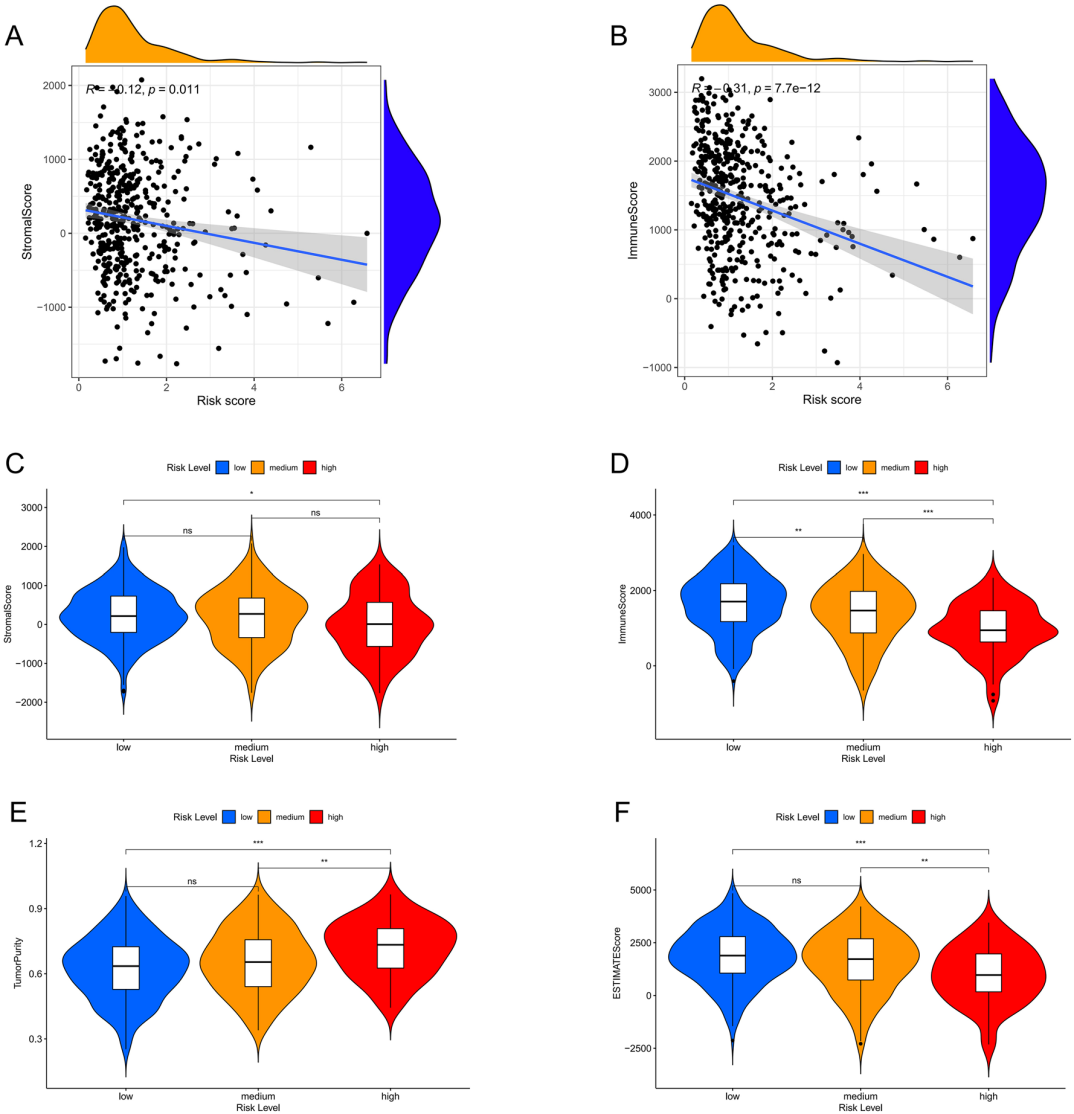
Correlation analysis between prognostic model and tumor microenvironment using R software. (**A**) Spearman analysis between stromal score and risk score. (**B**) Spearman analysis between immune cell score and risk score. (**C**–**F**) Differences in stromal score (**C**), immune scores (**D**), tumor purity (**E**), estimate score (**F**) between risk groups. *p-value < 0.05, **p-value < 0.01, ***p-value < 0.001.

Tumor microenvironment was quantified by score of stromal cells (stromal score) and score of immune cells (immune score). Sum of immune score and stromal score equals the tumor microenvironment (estimate score). Spearman analysis showed that Immune score, stromal score and estimate score were negatively correlated to risk score. Tumor purity was positively correlated with risk score (Fig. 6). There was a positive correlation between tumor mutation burden (TMB) and risk score (Supple Fig. 4E). In the waterfall plot, the proportion of samples with mutations in the high-risk group was significantly higher than that in the low-risk group, and missense mutations were the most common type of mutation. The most frequently mutated gene was TP53, which was also the gene with the most significant difference in mutation ratio among the high- and low-risk group, followed by TTN (Supple Fig. 4A–C). TP53 gene and KRAS gene have mutually exclusive mutation relationship (Supple Fig. 4D).

### Correlation analysis between prognostic model and immune

**Figure 7:**
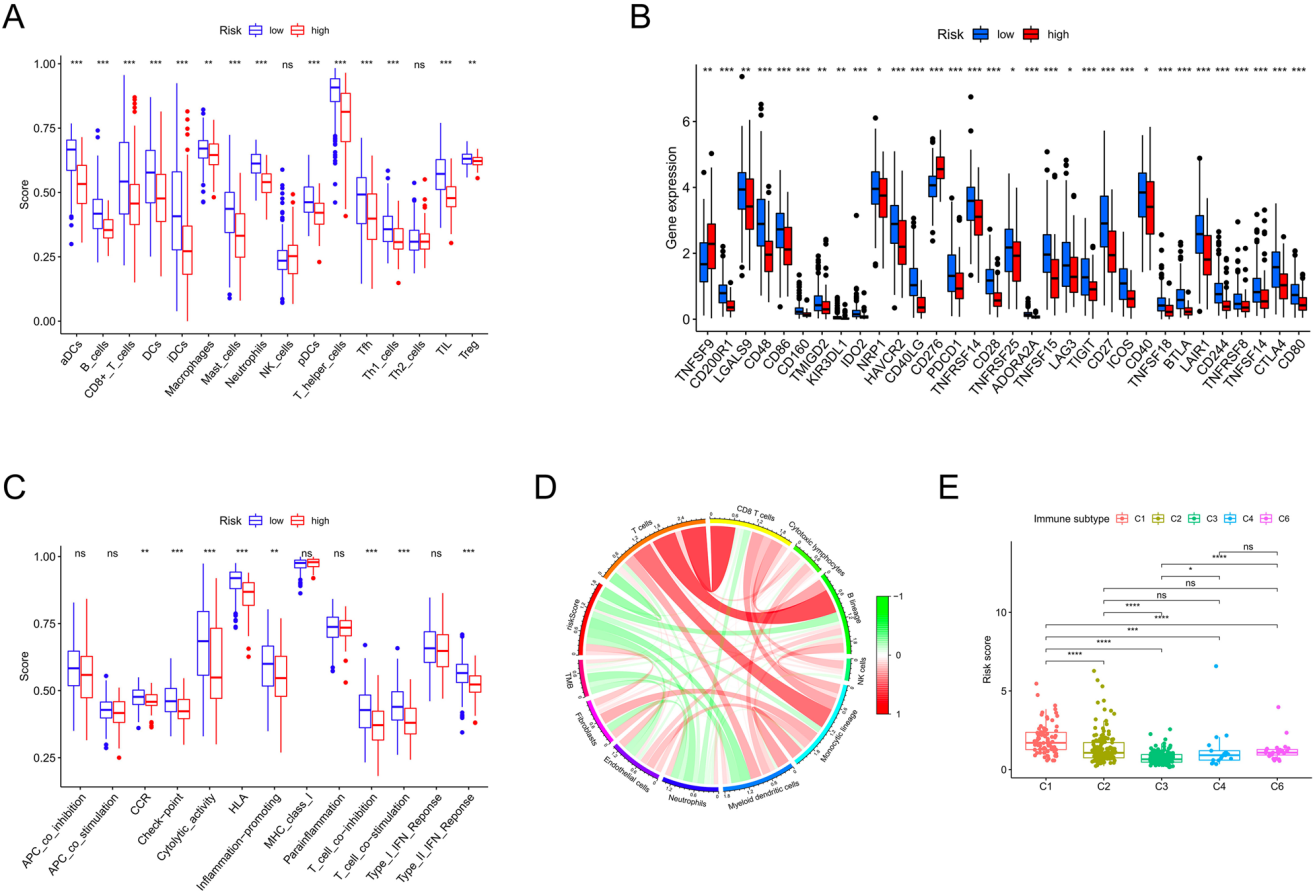
Correlation analysis between prognostic model and immune using R software. (**A**) Differences in 16 immune infiltration cells between high- and low-risk groups. (**B**) Differences in gene expression level of immune checkpoint-related genes between high- and low-risk patients. (**C**) Differences in 13 immune-related function between high- and low-risk groups. (**D**) Relationship among estimated infiltration abundances of immune cells, risk score and TMB. (**E**) Differences in risk scores of different immune subtypes. *p-value < 0.05, **p-value < 0.01, ***p-value < 0.001.

In order to further explore the correlation between risk score and immune status, 16 kinds of immune cells and related immune functions in all samples were scored by ssGSEA. Most of the estimated infiltration abundances of immune cells in the samples of high-risk group were down-regulated with statistical significance (*p*-value < 0.05) (Fig. 7A), including B cells, dendritic cells (DCs), immature dendritic cells (iDCs), activated dendritic cells (aDCs), plasmacytoid dendritic cells (pDCs), CD8^+^ T cells, macrophages, mast cells, neutrophils, T helper cells, T follicular helper cell (Tfh), Type 1 T help Cells (Th1 cells), tumor infiltrating lymphocyte (TIL), regulatory T cells (Treg). Both risk score and TMB were negatively correlated with abundance of multiple types of immune cells (Fig. 7D). In terms of immune function, the activity scores of the samples in high-risk group were significantly lower in check-point, cytolytic activity, Human lymphocyte antigen (HLA)inflammation-promoting, T cell co-inhibition, T cell co-stimulation, type II interferon (IFN) response (Fig. 7C). CIBERSORT method was used to calculate the relative content of 22 types of immune cells in each sample. Similar to the result of ssGSEA, the relative content of B memory cells, resting CD4 T memory cells, monocytes, resting dendritic cells, mast cells were higher in the samples of the low-risk group, macrophage M0 is an exception, which is higher in the samples of high-risk group (Supple Fig. 5). Correlation analysis showed that the expression values of most immune checkpoint related genes were up-regulated in the low-risk group, including PD-CD1 and BTLA, while TNFSF9 and CD276 were down-regulated in the low-risk group (*p*-value < 0.05) (Fig. 7B). Previous studies have summarized the immune infiltration patterns of various cancer types, which can be defined as six types, including: C1 (wound healing), C2 (INF-γ dominant), C3 (inflammatory), C4 (lymphocyte depleted), C5 (immunologically quiet), C6 (TGF-β dominant). In this study, the ensemble average of risk score of the sample with type C1 (wound healing) was the highest, and that of the sample with type C3 (inflammatory) was the lowest (Fig. 7E).

### Immunotherapy effect and drug sensitivity analysis

**Figure 8:**
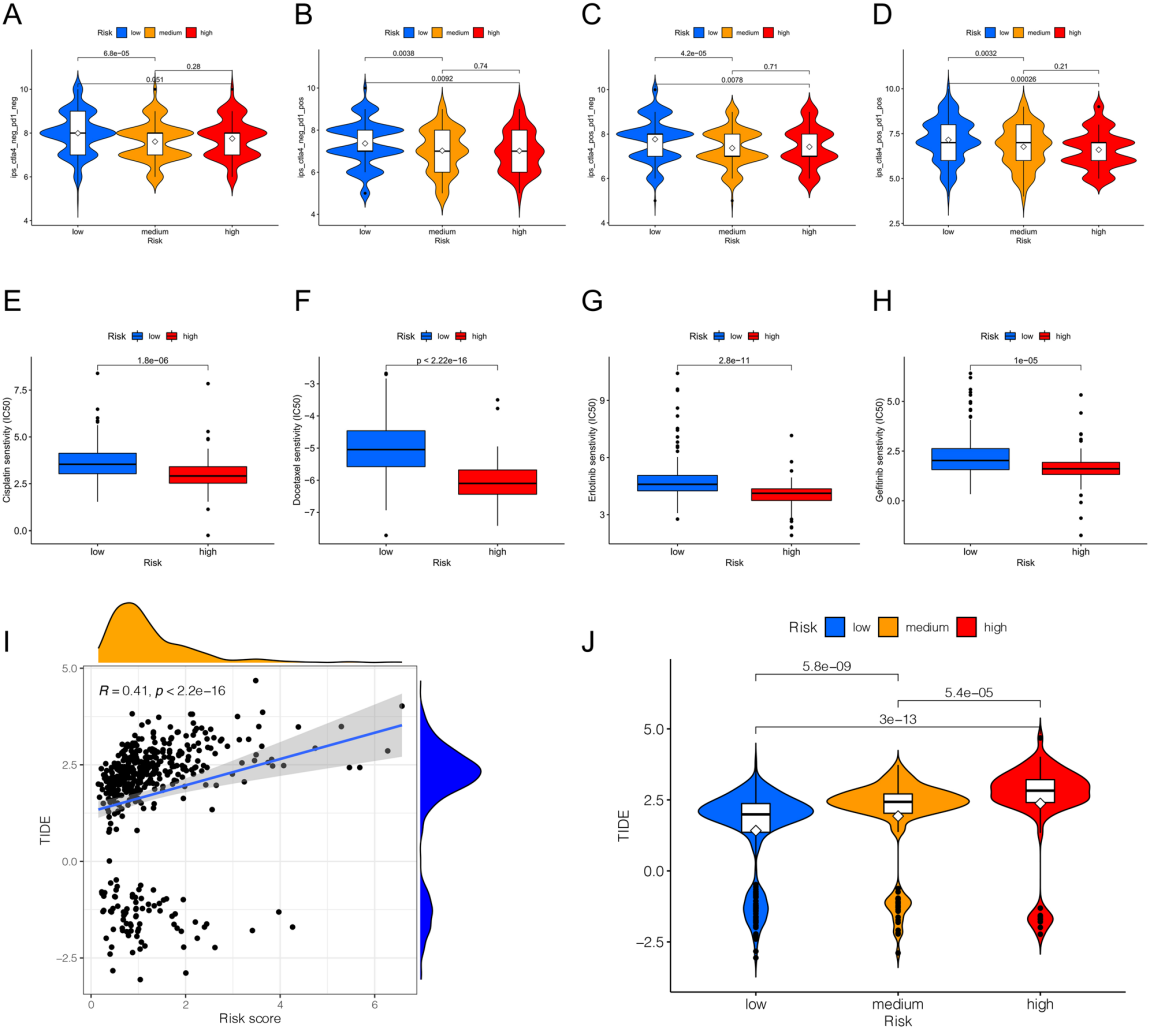
Immunotherapy effect and drug sensitivity analysis using R software. (**A**–**D**) Differences in IPS among different risk groups in the four situations: negative immunoresponse to both PD-L1/PD-1 inhibitors and CTLA-4 inhibitors (**A**); positive immunoresponse to PD-L1/PD-1 inhibitors (**B**); positive immunoresponse to CTLA-4 inhibitors (**C**); positive immunoresponse to both PD-L1/PD-1 inhibitors and CTLA-4 inhibitors (**D**). (**E**–**H**) Differences in sensitivity of cisplatin (**E**), docetaxel (**F**), erlotinib (**G**), gefitinib (**H**) between high and low risk groups. (**I**) Spearman analysis between TIDE score and risk score. (**J**) Differences in TIDE score among different risk groups. The hollow diamond patterns in the violin diagram represent the IPS or TIDE score ensemble average.

There was no statistical difference in immunophenoscore (IPS) between the high-risk and low-risk groups among samples that predicted a negative response in both immunotherapy regimens (Fig. 8A). Among tumor samples that were predicted to have a positive immune response to a single regimen of PD-1/PD-L1 (Fig. 8B) or CTLA4 inhibitors (Fig. 8C) and a positive immune response to both regimens (Fig. 8D), the ensemble average of IPS in the low-risk group were higher than those in the high-risk group, and the differences were statistically significant. Spearman analysis showed there is a positive correlation between the risk score and TIDE score (Fig. 8I). TIDE score increased with the grade of risk group (Fig. 8J). The results were statistically significant, suggesting that patients in the high-risk group may have the worst effect on PD-1/PD-L1 or CTLA-4 immune checkpoint inhibitors, while patients in the low-risk group may have the best effect. The 50% inhibiting concentration (IC50) of commonly used LUAD targeted drugs and chemotherapy drugs were calculated to analyze the drug sensitivity of 464 samples from TCGA. The IC50 of cisplatin (Fig. 8E), docetaxel (Fig. 8F), erlotinib (Fig. 8G) and gefitinib (Fig. 8H) were significantly increased in the low-risk group in TCGA, suggesting higher sensitivity of patients in the high-risk group to use these drugs.

## Discussion

In this study, we obtained inflammatory genes associated with prognosis of LUAD by univariate Cox analysis, and constructed a prognostic inflammation-related gene model by LASSO-penalized Cox regression. Stepwise regression and Akaike Information Criterion were used to determine the final model parameters to obtain the most suitable genes for inclusion in the model. A prognostic risk signature was generated based on eight inflammation-related genes, which can divide the samples into high-, medium- and low-risk group. The risk group could serve as an independent predictor for lung cancer prognosis, and participate in the construction of nomogram, which was successfully validated in an independent GEO dataset, indicating the general applicability of this signature.

Partial genes included in the prognostic model have been proved to be related to the occurrence and development of malignant tumors in current studies. CCL20 is expressed by macrophages, T cells and B cells and is responsible for the chemotactic attraction of immature dendritic cells, effector/memory T cells and B cells. It is up-regulated in tumor tissues and negatively correlated with the prognosis of lung cancer, thus it may be a potential therapeutic target^9^. CCR2 is one of the four chemokine receptors expressed by monocytes, it is related to the migration of inflammatory sites and plays a leading role in promoting the recruitment of monocytes with tumorigenic and metastatic activity^10,11^. ITGA5 is a member of the integrin α chain family, which participates in a variety of biological functions, including cell proliferation, differentiation, adhesion, survival and apoptosis^12^. It may also be involved in the PI3K/Akt signaling pathway that mediates inflammation, apoptosis and reactive oxygen species production of pulmonary endothelial cells^13–15^. PCDH7 is a cell surface receptor protein that is highly expressed in NSCLC, inducing cell transformation and promoting tumor growth in vitro and in vivo. The mitogen-activated protein kinase (MAPK) pathway plays a central role in PCDH7-mediated tumorigenesis^16^. In addition, the deletion of PCDH7 has been shown to increase the sensitivity of mutant lung cancer cells with KRAS mutation to MEK and ERK inhibitors, thus the treatment of PCDH7 may have a synergistic effect with EGFR or MAPK inhibitors^17^. NMI is an important component of a transcription factor complex that allows for the continuous activation of telomerase in breast and ovarian cancer, and participates in the regulation of bradykinin BDKRB2 and MAPK/ERK pathways, thus mediating tumor progression and metastasis^18^. GNAI3 is a potential tumor suppressor, which inhibits GNAI2-mediated myeloid-derived suppressor cells (MDSCs) proliferation and Colitis-Associated tumorigenesis by negatively regulating IL6 signaling pathway^19^. SLC11A2, a member of the SLC11 family, also known as DMT1, has been shown to control the iron pool in the cytoplasm, thus affecting the step of drug activation or the level of hydroxyl radicals in cells^20,21^. PSEN1 is a recognized gene associated with Alzheimer's disease, which can induce the intramembrane division of Notch receptors and then activate Notch signaling pathways. A study shows that its abnormal expression may activate downstream Notch1 signaling pathways and be related to the development of lung cancer^22–24^.

The mRNAs associated with high-risk group was enriched in common tumor-related pathways and functions, such as cell cycle, DNA replication, chromosome recombination, and cell desmosomes. While the mRNAs associated with low-risk group was enriched in T and B cell receptor signal pathways, chemokine signal pathways and so on, it also enriched in immune and inflammatory functions such as immune response, cytokine activity, regulation of T and B cells etc. The results suggest that low-risk group samples may be more active in immune and inflammation-related biological processes. While the high-risk group had higher tumor purity but lower stroma score and immune score, consistent with our previous analysis.

Immune cell infiltration was an important regulator of tumor progression, our study further found that there was a significant correlation between risk signature and immune status. The results of ssGSEA showed that the estimated infiltration abundances of most immune cells and activity scores of immune functions were higher in the low-risk group than in the high-risk group. The relative content of each type of immune cell in a single sample obtained using CIBERSORT method also showed a higher degree of immune infiltration in the low-risk group. The results suggest that the low-risk score may represent a more immunoreactive microenvironment and the high-risk score represents an immunosuppressed microenvironment, which may be one of the reasons for the poor prognosis of patients in the high-risk group. Previous literature suggest that inflammatory mediators and cytokines produced in the process of inflammatory response can establish gradients to recruit or reject immune cell subsets, and ultimately promote the formation of immunosuppressive microenvironment and immune escape, thus accelerating tumor metastasis^25,26^. Therefore, some studies have pointed out that the evaluation of tumor immune cell infiltration in tumor microenvironment suggests that "hot" or inflammatory tumors tend to have a better therapeutic response^27^. However, macrophage M0 infiltration levels were significantly increased in the high-risk group according to CIBERSORT. Macrophages M0 are nonactivated cells and belong to the different states of macrophages (M1, M2, M0), currently there are not enough clinical trials or research to prove that M0 is related to the occurrence, development and prognosis of lung cancer. The reasons and mechanisms for the case are still unclear, which may require more studies or experiments to explore.

According to the six pan-cancer immunophenotypes summarized in previous studies, type C3 (inflammatory) is characterized by immune control and immune balance, and has the best prognosis among the six types, while type C1 (wound healing) is characterized by increased expression of angiogenic genes and high tumor cell proliferation rate, suggesting a poor prognosis. In this study, we found that the risk score of type C3 was the lowest, while that of type C1 was the highest, which was consistent with the difference in prognosis predicted by our model.

Tumor mutation burden (TMB) is defined as the total number of somatic/acquired mutations in each coding region of the tumor genome (Mut/Mb)^28^. In this study, risk scores were positively correlated with TMB, with a higher proportion of mutation events occurring in tumor samples in the high-risk group. Most of the mutant genes are mainly missense mutations, which may produce new antigens recognized by the host immune system and lead to anti-tumor immune response. tumors with high mutation burden may produce more new antigens, thus increasing the possibility of immune recognition and tumor cell killing^29^. However, due to the instability and heterogeneity of TMB in tissues and the lack of unified standard detection methods, the effect of immunotherapy could not be accurately predicted^30–32^.

Interestingly, we found significant differences in the expression of multiple immune checkpoint genes in different risk groups, suggesting that immunotherapy effect may be associated with risk group, and different immune checkpoint inhibitors may suit to different risk groups. To further investigate the effects of immunotherapy in different groups, we analyzed IPS of LUAD from the TCGA cohort on the TCIA website. IPS is comprehensive score used to predict the effects of immunotherapy based on representative immune cell characteristics and tumor genotypes. The analysis showed that the ensemble averages of IPS in the low-risk group samples with positive immunoresponse to PD-L1/PD-1 or CTLA-4 inhibitors was higher than that in the high-risk group. What's more, another immune score from Immune Dysfunction and Exclusion (TIDE), a computational architecture designed by Peng Jiang et al. based on tumor immune escape mechanism, appears the same result^33^. The study involved more than 30,000 samples, and the results are also considered to be able to replace a single biomarker to effectively predict the effect of immune checkpoint inhibitor therapy. A high TIDE score in the sample indicated poor efficacy of immune checkpoint blocking therapy (ICB) and short survival after ICB treatment. In our study, TIDE score was positively correlated with risk score, and the score of low-risk group was significantly lower than that of high-risk group. Combined with IPS and TIDE, patients in the low-risk group may have a better immune response to immunotherapy regiments, and the risk signature has the potential to screen LUAD patients who are immunogenic and more responsive to ICIs.

In addition, current studies have shown that inflammation may be associated with tumor multidrug resistance, and the common mechanisms of tumor drug resistance, such as the overexpression of membrane-anchored MDR transporters, can be directly affected by inflammation and inflammatory mediators^34^. Taking a single gene as an example, previous studies have shown that the increased expression of inflammation-related gene COX-2 is related to the level of MDR protein, which can strongly interfere with the results of chemotherapy in cancer patient, suggesting that inflammatory genes may be used as a predictor of tumor treatment efficacy^35^. In our study, tyrosine kinase inhibitors (TKIs) with epidermal growth factor receptor (EGFR) as target genes, such as gefitinib and erlotinib, and commonly used chemotherapy drugs, such as cisplatin and docetaxel, were more sensitive in high-risk group of patients. This result has certain reference significance for anti-tumor drug strategy.

There are some limitations in our study. Prediction results of prognosis by model and guidance results of drug use by risk signature still needs to be further verified in clinical studies with large samples. In addition, the prognosis prediction and subsequent analysis in our study were based on the data from RNA-Seq in TCGA, thus our model only applies to the data based on RNA-Seq, which belongs to the technology Next Generation Sequence (NGS). Raw data “HTSeq-Counts” of the patient's lung adenocarcinoma tissues provided by RNA-Seq should be standardized by FPKM method to acquire the gene expression values, then the formula provided by the prognostic model in the methodological section of our study can be used to calculate the risk score and identify risk groups for LUAD patients (low risk < 1.05, 1.05 ≤ medium risk ≤ 2.01, high risk > 2.01).

## Conclusion

In conclusion, our study elaborated the prognostic significance of important inflammation-related genes in patients with lung adenocarcinoma, and constructed a prognostic model composed of eight genes, which can accurately predict the prognosis of patients with lung adenocarcinoma. This study will help us further understand the role of inflammation-related genes in influencing cellular pathways, immune cell infiltration, immune checkpoint gene expression, tumor mutation, tumor microenvironment and antitumor drug selection. This study is helpful to guide more effective immunotherapy strategies for lung adenocarcinoma.

## Materials and methods

### Data acquisition and document preparation

RNA-seq profiles of LUAD patients were downloaded from the TCGA (https://portal.gdc.cancer.gov/), including fragments per kilobase per million (FPKM). The workflow type is HTSeq-FPKM. R-package “limma” was used to average the repeated data of RNA expression profile. Clinical data of LUAD samples were retrieved and downloaded from TCGA, including gender, age, tumor stage and survival information. Tumor samples were distinguished from normal samples by TCGA ID. In the construction of the gene prognosis model, only tumor samples and samples with complete clinical information about survival time and survival status were included. During the subsequent construction of clinically relevant prognostic nomogram, samples with complete information were retained according to the clinical factors included in the nomogram. The dataset GSE30219 was downloaded from GEO (https://www.ncbi.nlm.nih.gov/geo/) for verification, chip platform is based on Affymetrix Human Genome U133 Plus 2.0 Array. The download content includes the GSE30219 series matrix file and the annotated file “GPL570”. Inflammation-related genes were obtained from Molecular Signatures Database (MSigDB) on GSEA website (https://www.gsea-msigdb.org/gsea/msigdb/index.jsp). Data related to immunophenoscore of PD-1/PD-L1 and CTLA-4 immunotherapy in LUAD cohort were obtained from TCIA (https://tcia.at/home). Tumor Immune Dysfunction and Exclusion (TIDE) score of LUAD were acquired from TIDE website (https://tide.dfci.harvard.edu/) according to RNA-Seq in TCGA. Statistical analyses and the map of figures were performed using R software (version 4.0.5, https://cran.r-project.org/), X-tile software (version 1.0.2643.21670, https://medicine.yale.edu/lab/rimm/research/software/) and GSEA software (version 4.1, http://www.gsea-msigdb.org/gsea/downloads.jsp). All data obtained from TCGA, GEO, GSEA, TCIA and TIDE are public, the involved human data in TCGA and GEO databases have obtained ethical approval. Our study based on public source and the acquisition process follows the rules and guidelines of the official website, there are no ethical issues and other conflicts of interests.

### Obtain prognostic inflammation-related genes and construct genetic prognostic model

Univariate Cox regression analysis was used to obtain inflammation-related genes associated with OS. LASSO regression algorithm in R-package “glmnet” was used to establish the penalty coefficient and selection variables, and the ten-fold cross-validation method was used to determine the penalty coefficient (λ) of the regression model. By applying the penalty coefficient to shrink the regression coefficient of prognosis-related inflammatory genes, most of the independent variable coefficients were reduced to zero, and only a relatively small number of genes with non-zero weight were retained. After obtaining the optimal number of variables, the multivariate Cox regression analysis was further carried out. In this study, we used Akaike Information Criterion (AIC) to complete the parameter selection of multivariate Cox proportional hazard model. The AIC is developed by Hirotugu Akaike in 1974^36^. It is a weighting function of fitting accuracy and number of parameters: AIC = 2K − 2ln(L). "K" represents the number of estimated parameters, "L" represents the maximum value of likelihood function for this model. When AIC value is the minimum, this model can best fit data and contain the least free parameters. We used the "Coxph" function in R software for multivariate regression analysis, and the "step" function carried out the forward and backward stepwise regression method to screen parameters. In this process, by adjusting the number of model parameters, the multivariate Cox proportional hazard model with minimum AIC value was obtained.

### Risk groups and genetic prognostic model validation

The model gene's regression coefficient is multiplied by the gene's expression value and summed up to give each patient's risk score. The formula for calculating the risk score is as follows: Risk Score = (Gene 1 Expression × Coefficient) + (Gene 2 Expression × Coefficient) + ⋯ + (Gene n Expression × Coefficient). "Gene n Expression" represents the expression values of gene included in the regression model, "Coefficient" represents the regression coefficient of the gene. X-tile software was used to obtain the best cut-off value of risk score^37^. The software uses Kaplan–Meier method and log-rank test to analyze and compare the survival differences among risk groups under different cut-off values. When the *p*-value is minimum, the risk score is the best cut-off value, which can divide samples into different risk groups. PCA and UMAP were performed using the function "prcomp" and R-package "UMAP" in R software. R-package "Survminer" was used for survival analysis, Kaplan–Meier method was used to evaluate the survival difference of patients in different risk groups, log-rank test was used for inter-group comparison, the result was considered to be statistically significant when *p*-value < 0.05. The "time ROC" R packet is used to construct a time-dependent receiver operating characteristic (ROC) curve to measure the prediction performance of the model. When the area under the curve (AUC) is greater than 0.6, it is considered to have prediction ability. Univariate and multivariate Cox analyses were used to identify the prognostic significance of risk score and clinical characteristics. Bootstrap re-sampling method was used to calculate the C-index of risk score and clinical characteristics in the univariate regression model to compare the difference in their predictive ability. The risk signature and prognostic model performance were verified by independent data set GSE30219 from GEO.

### Construct and verify the prognostic nomogram

The prognostic nomogram was constructed by using the R-package "rms" in R software by integrating risk group and clinical characteristics. Each factor included in the nomogram corresponds to an axis used for scoring, and the survival probability of the patient in 1, 3 and 5 years can be obtained according to the sum of the scores of all the prognostic factors in the nomogram. Calibration curves and C-index were used to evaluate the nomogram performance. We used bootstrap re-sampling method to re-sample for 1000 times and draw calibration curves to reflect the consistency between the predicted probability and the observed probability in a visual form. When the predicted probability is equal to the observed probability, the calibration curve generated will coincide with the 45° diagonal line emitted along the diagonals in the graph. External validation of Nomogram is performed using the dataset GSE30219 from GEO.

### Gene set enrichment analysis

The pathways and functions enrichment analysis for the mRNAs associated with the high or low risk was carried out using c2.cp.kegg.v7.4.symbols.gmt and c5.go.v7.4.symbols.gmt as gene sets database at 1,000 random sample permutations using GSEA software^38–42^.The enrichment functions or pathways were statistically significant when *p*-value < 0.05.

### Tumor microenvironment, tumor mutation burden, immune infiltration

We used the R-package "ESTIMATE" to calculate the immune and stromal scores and tumor purity scores for the samples, quantify the levels of immune and stromal cell infiltration in different risk groups, and compare differences in the tumor microenvironment. To explore the correlation between risk groups and TMB, we analyzed the available somatic mutation data from LUAD cohort in TCGA, which was downloaded in MAF format and was analyzed using R-package “maftools”. We used single-sample gene set enrichment analysis (ssGSEA) to calculate the scores of 16 immune cells and 13 immune-related pathways in each sample using a "GSVA" R-package^43,44^. CIBERSORT method is an excellent tool for assessing immune cell infiltration and can be used to assess the relative abundances of 22 types of immune cells in single sample^45^. These two methods were combined to compare the differences in immune cell infiltration degree or functional activity between different risk groups. Furthermore, we compared the difference in the ensemble average of risk score between different immunophenotypes, which were identified by previous literature^46^. The immunophenotyping data of pan-cancer samples were obtained from the NCI Genomic Data Commons official website (GDC, https://portal.gdc.cancer.gov), and were intermixed with LUAD samples of TCGA. According to the immune checkpoint-related genes summarized in previous literature, the differences in the ensemble average of expression values of immune checkpoint-related genes among different risk groups were compared. Analysis of variance (ANOVA) was used for comparison between different groups. Spearman analysis was used for correlation analysis (the correlation between TMB and risk score as an example). The results considered to have statistical significance when *p*-value < 0.05.

### Immunotherapy effect and drug sensitivity

Immunophenoscore (IPS) is a score calculated quantitatively based on MHC molecules, immune regulators, effector cells and suppressor cells. It is a good predictor of the response of immune checkpoint inhibitors (ICIs). IPS is calculated based on representative cell type gene expression, with scores ranging from 0 to 10. The IPS of each LUAD patient was obtained from TCIA (https://tcia.at/home)^44,47^. TIDE is a computational framework based on two major mechanisms of tumor immune escape, and provide predictive results for immunotherapy. TIDE score of LUAD cohort in TCGA database were acquired from TIDE website. We compared the predictive efficacy of immunotherapy in different risk groups by comparing IPS and TIDE scores. The "pRRophetic" algorithm in R software was used to estimate the IC50 value of commonly used targeted drugs and chemotherapy drugs for lung cancer, and the drug sensitivity differences among different risk groups were compared. ANOVA was used to compare the differences in the ensemble average of different risk groups in IPS score, TIDE score and IC50 value, the results considered to have statistical significance when *p*-value < 0.05.

## Supplementary Information

Supplementary Figure 1.

Supplementary Figure 2.

Supplementary Figure 3.

Supplementary Figure 4.

Supplementary Figure 5.

Supplementary Table 1.

Supplementary Table 2.

Supplementary Table 3.
