# Supplementary figures and images for: Identification and validation of a novel prognostic model of inflammation-related gene signature of lung adenocarcinoma
Source: Sci Rep. 2022 Aug 30;12:14729. doi: 10.1038/s41598-022-19105-8 (PMC9427773; doi:10.1038/s41598-022-19105-8)

Supplementary Figure 1

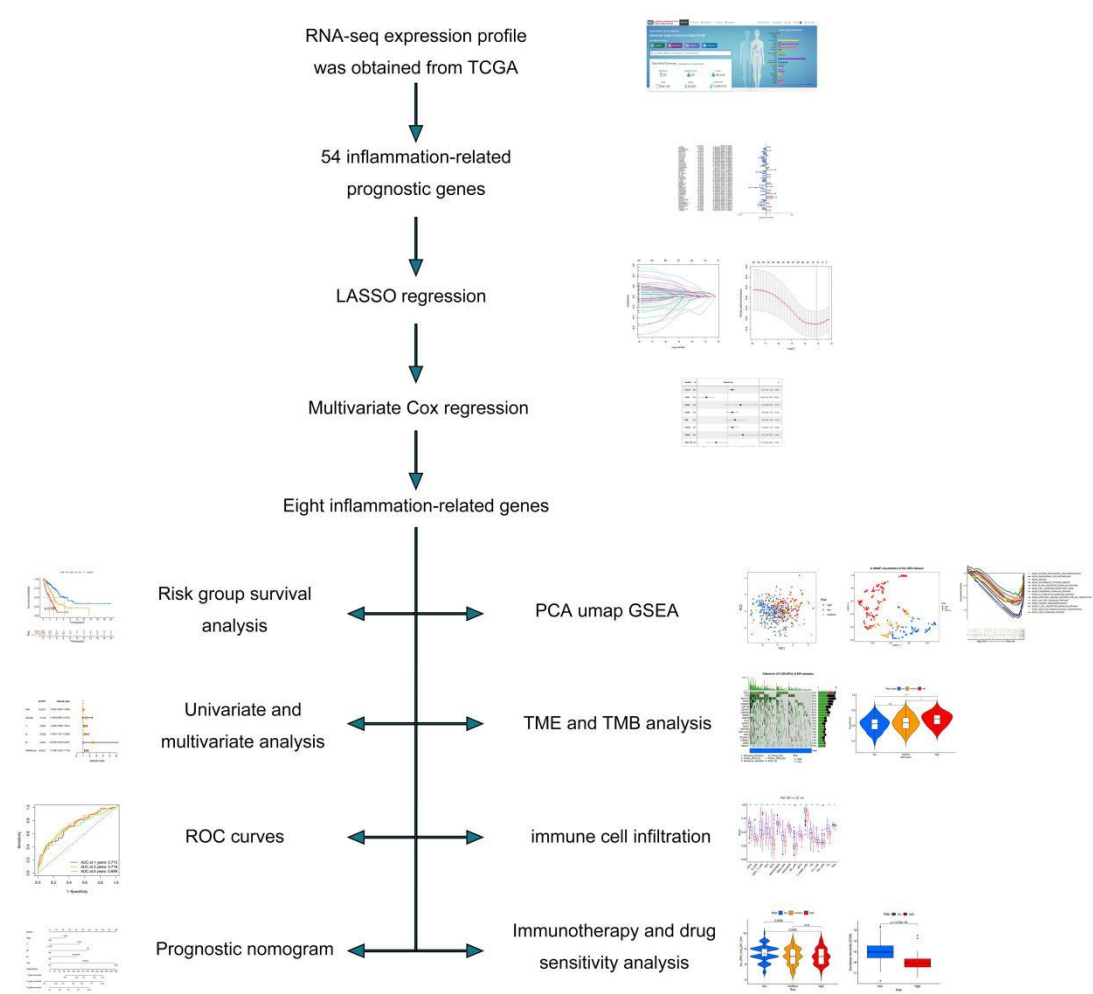

Supplementary Figure 1. Flow chart of the study.

Supplement: Supplementary file 1 — Supplementary Figure 1. [file 41598_2022_19105_MOESM1_ESM.pdf]
