# Supplementary material for: Identification and validation of a novel prognostic model of inflammation-related gene signature of lung adenocarcinoma
Source: Sci Rep. 2022 Aug 30;12:14729. doi: 10.1038/s41598-022-19105-8 (PMC9427773; doi:10.1038/s41598-022-19105-8)
Supplement: Supplementary file 2 — Supplementary Figure 2. [file 41598_2022_19105_MOESM2_ESM.pdf]

Supplementary Figure 2

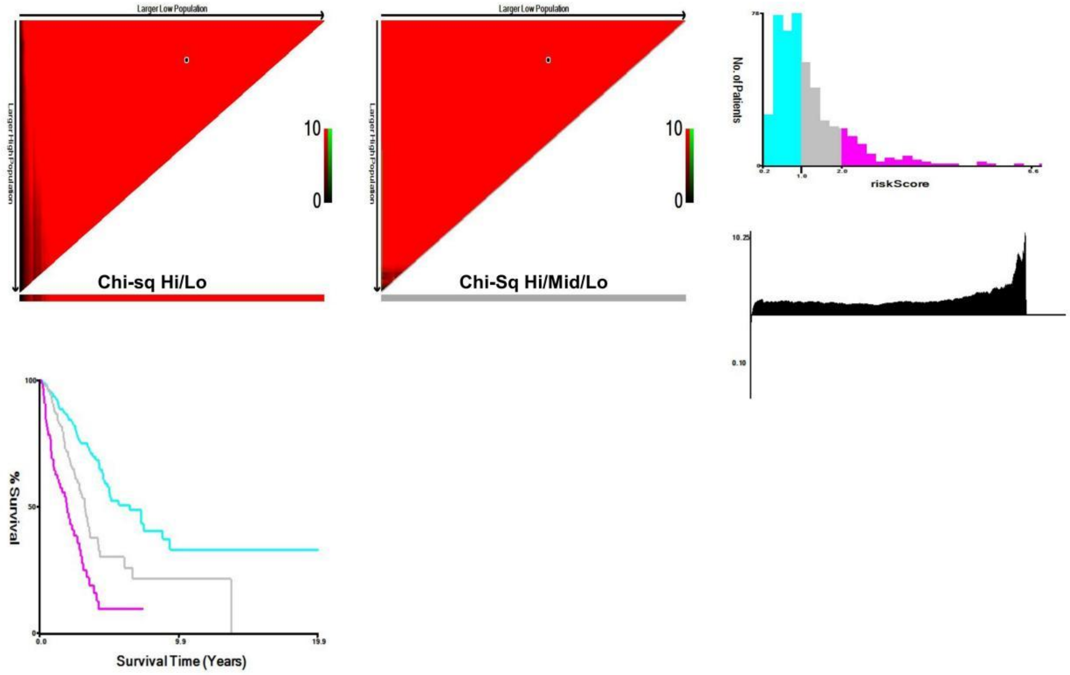

Subpopulation Cutpoints:

Pt No	% Total	Events	Rate	Rank	Range
255	54.96	69	27.06	0 to 254	0.15 thru 1.05
142	30.60	61	42.96	255 to 396	1.05 thru 2.01
67	14.44	46	68.66	397 to 463	2.01 thru 6.58
464	100.00	176	37.93	0 to 463	0.15 thru 6.58

Statistics:

Variable	Value	
Chi-Sq Hi/Mid/Lo	66.9960	Max: 66.9960
Lo vs Mid	17.9738	
Mid vs Hi	14.9034	
Lo vs Hi	67.5664	
Relative Risk 1 vs 2 vs 3	1.00 / 1.59 / 2.54	

Supplementary Figure 2. The process of obtaining the best cut-off values in the TCGA cohort using X-Tile software.
