# Supplementary material for: Identification and validation of a novel prognostic model of inflammation-related gene signature of lung adenocarcinoma
Source: Sci Rep. 2022 Aug 30;12:14729. doi: 10.1038/s41598-022-19105-8 (PMC9427773; doi:10.1038/s41598-022-19105-8)
Supplement: Supplementary file 3 — Supplementary Figure 3. [file 41598_2022_19105_MOESM3_ESM.pdf]

Supplementary Figure 3

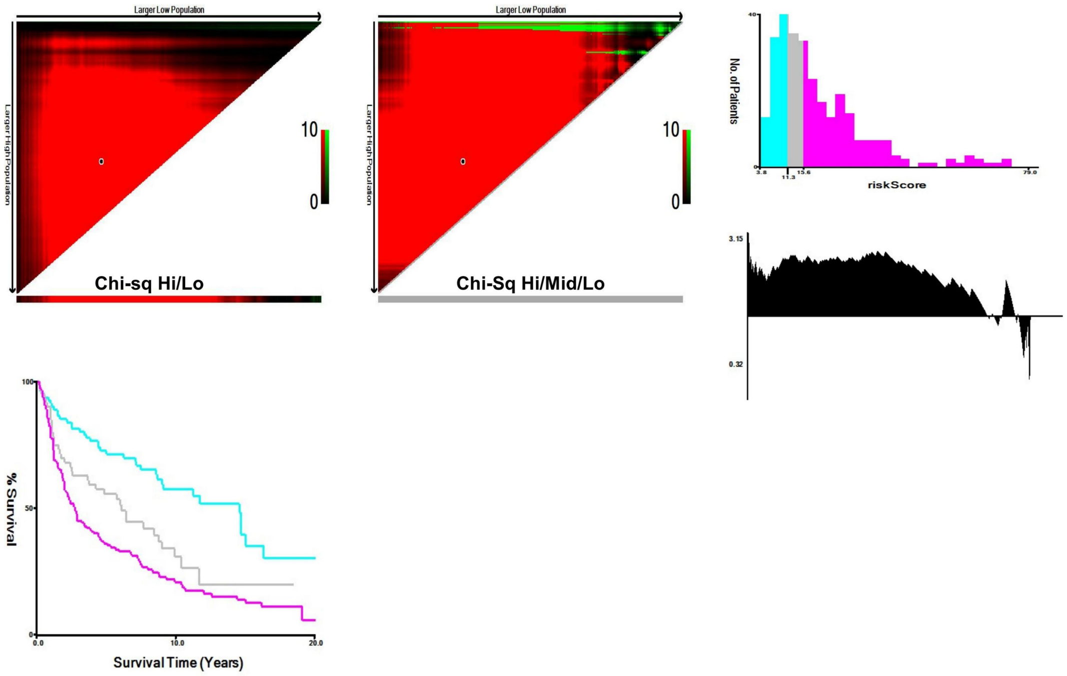

Subpopulation Cutpoints:

Pt No	% Total	Events	Rate	Rank	Range
81	28.03	38	46.91	0 to 80	3.77 thru 11.27
60	20.76	38	63.33	81 to 140	11.28 thru 15.63
148	51.21	120	81.08	141 to 288	15.70 thru 78.98
289	100.00	196	67.82	0 to 288	3.77 thru 78.98

Statistics:

Variable	Value	
Chi-Sq Hi/Mid/Lo	30.0865	Max: 34.1336
Lo vs Mid	8.1384	
Mid vs Hi	3.9879	
Lo vs Hi	29.3453	
Relative Risk 1 vs 2 vs 3	1.00 / 1.35 / 1.73	

Supplementary Figure 3. The process of obtaining the best cut-off values in GSE30219 using X-Tile software.
