# Supplementary material for: Identification and validation of a novel prognostic model of inflammation-related gene signature of lung adenocarcinoma
Source: Sci Rep. 2022 Aug 30;12:14729. doi: 10.1038/s41598-022-19105-8 (PMC9427773; doi:10.1038/s41598-022-19105-8)
Supplement: Supplementary file 4 — Supplementary Figure 4. [file 41598_2022_19105_MOESM4_ESM.pdf]

Supplementary Figure 4

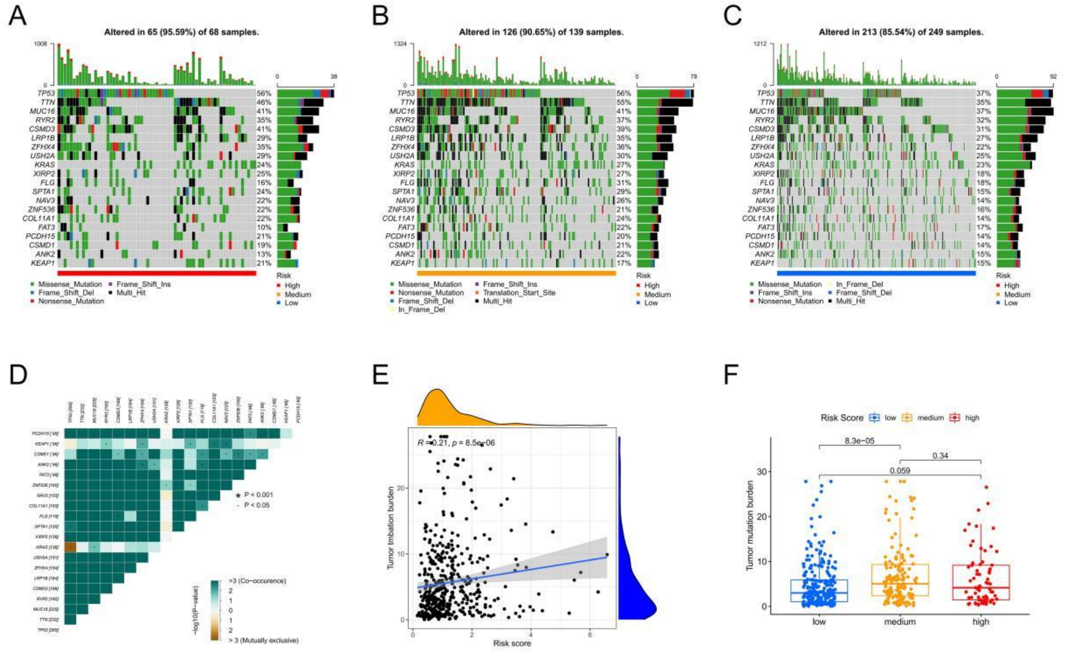

Supplementary Figure 4. Correlation analysis between prognostic model and TMB using R software.

(A, B, C) The waterfall plots showing the differences of tumor somatic genomic mutation in high- **(A)**, medium- **(B)** and low-risk group **(C)**. **(D)** Correlation of mutated genes. **(E)** Relationships between risk scores and TMB. **(F)** Comparison of TBM between different risk groups.
