# Supplementary material for: Identification and validation of a novel prognostic model of inflammation-related gene signature of lung adenocarcinoma
Source: Sci Rep. 2022 Aug 30;12:14729. doi: 10.1038/s41598-022-19105-8 (PMC9427773; doi:10.1038/s41598-022-19105-8)
Supplement: Supplementary file 5 — Supplementary Figure 5. [file 41598_2022_19105_MOESM5_ESM.pdf]

Supplementary Figure 5

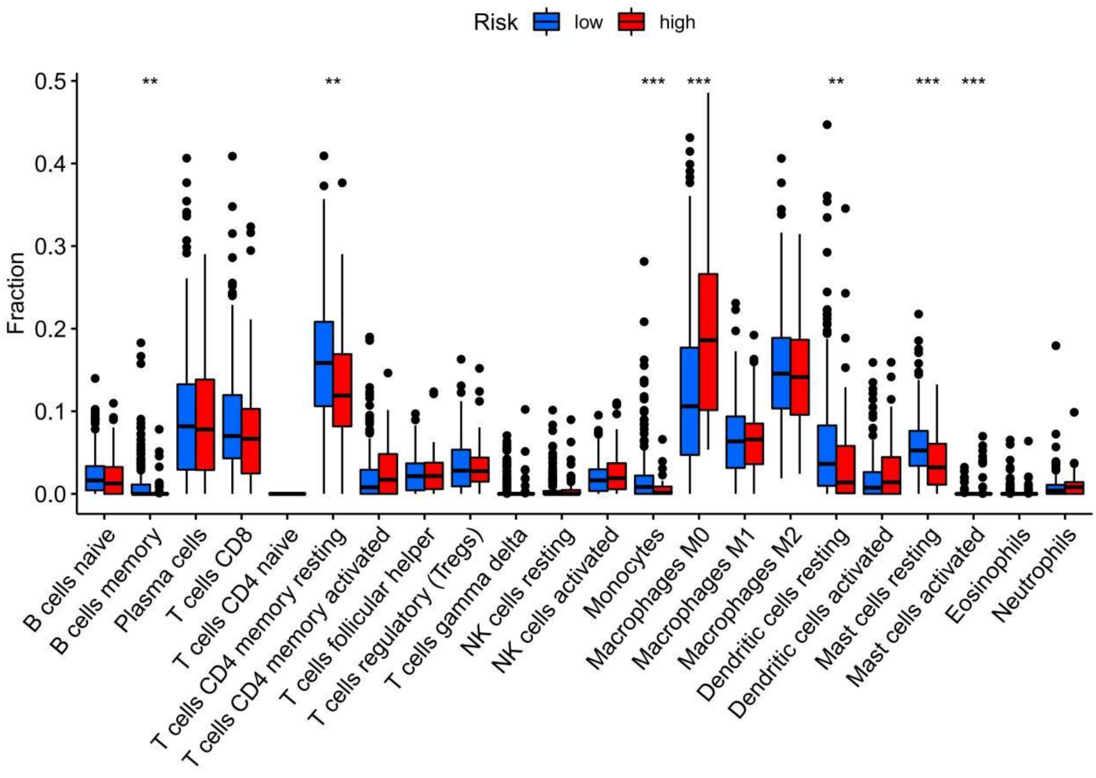

Supplementary Figure 5. The relative contents of 22 immune cell infiltration calculated by CIBERSORT method using R software. * p-value < 0.05, ** p-value < 0.01, *** p-value < 0.001.
