# Supplementary material for: Identification and validation of a novel prognostic model of inflammation-related gene signature of lung adenocarcinoma
Source: Sci Rep. 2022 Aug 30;12:14729. doi: 10.1038/s41598-022-19105-8 (PMC9427773; doi:10.1038/s41598-022-19105-8)
Supplement: Supplementary file 6 — Supplementary Table 1. [file 41598_2022_19105_MOESM6_ESM.pdf]

Supplementary Table 1

Eight inflammation-related genes of prognostic model.

Gene	Coefficient	HR	HR.95L	HR.95H	*p*-value
**CCL20**	0.1241	1.1322	1.0463	1.2251	0.0020
**CCR2**	-0.6192	0.5384	0.4315	0.6717	0.0000
**GNAI3**	0.3660	1.4419	0.8976	2.3164	0.1302
**ITGA5**	0.1239	1.1319	0.9633	1.3301	0.1323
**NMI**	0.2050	1.2275	0.9484	1.5887	0.1194
**PCDH7**	0.1330	1.1423	0.9794	1.3322	0.0901
**PSEN1**	0.4300	1.5373	1.0000	2.3632	0.0500
**SLC11A2**	-0.3382	0.7131	0.5423	0.9375	0.0154
