# Supplementary material for: Identification and validation of a novel prognostic model of inflammation-related gene signature of lung adenocarcinoma
Source: Sci Rep. 2022 Aug 30;12:14729. doi: 10.1038/s41598-022-19105-8 (PMC9427773; doi:10.1038/s41598-022-19105-8)
Supplement: Supplementary file 7 — Supplementary Table 2. [file 41598_2022_19105_MOESM7_ESM.pdf]

Supplementary Table 2

Clinical baseline characteristics table of TCGA cohort in different risk groups.

	Low Risk (N = 170)	Medium Risk (N = 88)	High Risk (N = 55)	Total (N = 313)
**Gender**				
FEMALE	91 (53.5%)	43 (48.9%)	28 (50.9%)	162 (51.8%)
MALE	79 (46.5%)	45 (51.1%)	27 (49.1%)	151 (48.2%)
**Age (years)**				
Mean (SD)	65.1 (10.2)	63.7 (10.5)	64.1 (10.6)	64.5 (10.4)
Median [Min, Max]	67.0 [33.0, 86.0]	64.0 [38.0, 84.0]	65.0 [40.0, 85.0]	66.0 [33.0, 86.0]
**Stage**				
Stage I	105 (61.8%)	43 (48.9%)	15 (27.3%)	163 (52.1%)
Stage II	31 (18.2%)	25 (28.4%)	19 (34.5%)	75 (24.0%)
Stage III	22 (12.9%)	16 (18.2%)	17 (30.9%)	55 (17.6%)
Stage IV	12 (7.1%)	4 (4.5%)	4 (7.3%)	20 (6.4%)
**T**				
T1	67 (39.4%)	23 (26.1%)	6 (10.9%)	96 (30.7%)
T2	83 (48.8%)	56 (63.6%)	36 (65.5%)	175 (55.9%)
T3	8 (4.7%)	8 (9.1%)	9 (16.4%)	25 (8.0%)
T4	12 (7.1%)	1 (1.1%)	4 (7.3%)	17 (5.4%)
**M**				
M0	158 (92.9%)	84 (95.5%)	51 (92.7%)	293 (93.6%)
M1	12 (7.1%)	4 (4.5%)	4 (7.3%)	20 (6.4%)
**N**				
N0	125 (73.5%)	52 (59.1%)	23 (41.8%)	200 (63.9%)
N1	28 (16.5%)	19 (21.6%)	18 (32.7%)	65 (20.8%)
N2	16 (9.4%)	17 (19.3%)	14 (25.5%)	47 (15.0%)
N3	1 (0.6%)	0 (0%)	0 (0%)	1 (0.3%)
**Survival State**				
Alive	122 (71.8%)	46 (52.3%)	15 (27.3%)	183 (58.5%)
Dead	48 (28.2%)	42 (47.7%)	40 (72.7%)	130 (41.5%)
