# Supplementary material for: Identification and validation of a novel prognostic model of inflammation-related gene signature of lung adenocarcinoma
Source: Sci Rep. 2022 Aug 30;12:14729. doi: 10.1038/s41598-022-19105-8 (PMC9427773; doi:10.1038/s41598-022-19105-8)
Supplement: Supplementary file 8 — Supplementary Table 3. [file 41598_2022_19105_MOESM8_ESM.pdf]

Supplementary Table 3

Clinical baseline characteristics table of GSE30219 in different risk groups.

	Low Risk (N=79)	Medium Risk (N=58)	High Risk (N=143)	Total (N=280)
**Gender**				
Female	16 (20.3%)	13 (22.4%)	12 (8.4%)	41 (14.6%)
Male	63 (79.7%)	45 (77.6%)	131 (91.6%)	239 (85.4%)
**Age (years)**				
Mean (SD)	57.7 (15.4)	62.8 (9.41)	62.5 (9.02)	61.2 (11.4)
Median [Min, Max]	60.0 [15.0, 84.0]	63.5 [44.0, 80.0]	63.0 [41.0, 82.0]	62.0 [15.0, 84.0]
**T**				
T1	54 (68.4%)	34 (58.6%)	75 (52.4%)	163 (58.2%)
T2	18 (22.8%)	14 (24.1%)	33 (23.1%)	65 (23.2%)
T3	4 (5.1%)	5 (8.6%)	22 (15.4%)	31 (11.1%)
T4	3 (3.8%)	5 (8.6%)	13 (9.1%)	21 (7.5%)
**M**				
M0	77 (97.5%)	57 (98.3%)	141 (98.6%)	275 (98.2%)
M1	2 (2.5%)	1 (1.7%)	2 (1.4%)	5 (1.8%)
**N**				
N0	61 (77.2%)	41 (70.7%)	91 (63.6%)	193 (68.9%)
N1	10 (12.7%)	9 (15.5%)	32 (22.4%)	51 (18.2%)
N2	7 (8.9%)	5 (8.6%)	15 (10.5%)	27 (9.6%)
N3	1 (1.3%)	3 (5.2%)	5 (3.5%)	9 (3.2%)
**Survival State**				
Alive	43 (54.4%)	20 (34.5%)	28 (19.6%)	91 (32.5%)
Dead	36 (45.6%)	38 (65.5%)	115 (80.4%)	189 (67.5%)
